# Impact of Controlled Breathing Techniques in Virtual Reality Environments on the Psychological Status of Oral Cancer Patients: A Case Report

**DOI:** 10.7759/cureus.51366

**Published:** 2023-12-30

**Authors:** Neha M Chitlange, Vaishnavi Yadav

**Affiliations:** 1 Cardiovascular and Respiratory Physiotherapy, Ravi Nair Physiotherapy College, Datta Meghe Institute of Higher Education and Research, Wardha, IND

**Keywords:** controlled breathing, virtual reality, depression, anxiety, oral squamous cell carcinoma

## Abstract

Oral cancer is among the six most common cancers worldwide. It is associated with a poor prognosis, delayed clinical diagnosis, absence of a clear biomarker, and expensive treatment choices. It is a serious health risk. Among people with cancer, increased stress and low mood are prevalent psychological issues. One of the most intriguing new technologies for treating anxiety and depression in the clinical setting may be virtual reality (VR). VR has recently emerged as a fascinating remedy for many symptoms in place of conventional exposure-based treatments. Immersion in a virtual world created by a computer reduces avoidance and speeds up the processing of emotions. This case concerns a 40-year-old male who underwent a mandibulectomy for left buccal mucosa carcinoma. The most typical oral side effects following are discomfort, sepsis, difficulty in eating food, and talking difficulties. Pain and difficulty opening his mouth were his main complaints. Additionally, he could not move his tongue, making it challenging for him to speak and swallow. He had chewed tobacco for the previous 10 years. The patient was advised to engage in physiotherapy, which included jaw-opening exercises, gulping exercises, etc., to lessen his discomfort. And to reduce anxiety and stress, VR therapy was given to the patient. The case's findings indicate that all goals were achieved and the patient progressed in his daily activities compared to the outcome measures.

## Introduction

Malignant neoplasm is explained as any abandoned expansion of cells that enter and lead to the destruction of neighboring tissues [1]. Oral cancer manifests itself as a small, unusual, unresolved growth or pain in the mouthparts, which include the lips, cheeks, sinuses, tongue, hard and soft palate, and floor of the mouth, which extends to the oropharynx [1]. It is the sixth prevalent cancer worldwide [2]. India has the highest percentage of people with oral cancer and accounts for 30% of the total worldwide incidence of oral cancer [1]. Each year, approximately 77,000 fresh cases, along with 52,000 deaths, are documented in India, accounting for roughly one-fourth of worldwide incidences [2]. In India, approximately 70% of instances have been identified to be in severe stages. Oral squamous cell carcinoma, which accounts for 84-97% of all oral cancers, has a five-year survival rate of about 20%, indicating that delayed diagnosis leads to a virtually nonexistent chance of recovery [3]. Oral squamous cell carcinoma is frequently caused by potentially malignant tumors or typical epithelium linings [4]. Due to the high rate of betel nut use, buccal squamous cell carcinoma is common in Southeast Asian and Indian populations [5]. Tobacco use, in various forms such as chara, khaini, cigarettes, bidi, and hookah, has been identified as a significant contributor to dental tumors in young and older Indian populations [6].

Having trouble adjusting to the stress of life-threatening illnesses can be caused by anxiety and depression. For example, the span of hospitalization, treatment compliance, quality of life, and survival time for cancer patients are all jeopardized due to such issues [7]. Even though procedures are frequently necessary, they may hurt a patient's health. Following surgery, individuals might experience uncomfortable psychological side effects like anxiety, depression, and others [8]. These conditions may result in hemodynamic alterations and recovery delays [9].

According to recent studies, using virtual reality (VR) environments may increase the efficiency of controlled breathing techniques, which are known to be effective at reducing stress and anxiety [10]. VR, a rapidly evolving technology, can significantly change several aspects of our lives, including entertainment, education, and medical care. In the healthcare industry, VR can be used as an investigating tool for rehabilitation and even for relieving pain [11].

Controlled breathing exercises, also known as deep breathing or diaphragmatic breathing, emphasize actively managing your breathing patterns. These methods involve slowing down your breathing, taking deeper breaths both in and out, and focusing on the motion of your diaphragm [12]. By purposefully regulating your breath, controlled breathing techniques aim to calm the mind and reduce stress and anxiety. This is done to allow for the parasympathetic nervous system to be activated, which lessens the body's fight-or-flight response [13]. Controlling feelings like anxiety and tension can be facilitated by using controlled breathing techniques [14]. Although there are many different types of controlled breathing techniques, diaphragmatic breathing, relaxation technique, and pursed lip breathing are the most widely used [12].

## Case presentation

In this case, a 40-year-old man had a jaw ulcer in his lower left back that had not healed after four months. According to the patient, his ulcer started small and grew over time until it was about 4×3 cm in size, roughly oval-shaped, and reddish pink in color. The pain that gradually started was intermittent, dull aching, and localized and was accompanied by an inability to swallow solids and liquids. With this complaint, the patient first went to a private hospital, where incisional biopsy was performed. The biopsy revealed moderately differentiated squamous cell carcinoma, and he was referred to a super speciality cancer hospital. With the above complaint, the patient visited the hospital's outpatient department, where he gave a history of tobacco chewing for 10 years and claims that he quit the habit for five months. He also gave an account of weight loss of 5 kg in the past one month. Here, tests, such as computed tomography (CT) scans of the neck, paraspinal sinuses, and thorax as well as contrast-enhanced computed tomography (CECT) of the buccal cavity, were performed. The results showed grade 3 oral submucous fibrosis and carcinoma of the left buccal mucosa. He was admitted to the hospital on March 21, 2023, and underwent surgery in which composite resection of the lesion, segmental mandibulectomy from 31 to the angle of the mandible over the left side, alveolectomy from 26 to 28, modified radical neck dissection of the left side, and reconstruction with free fibula flap of the left side and nasolabial flap over the right side under general anesthesia were done on March 31, 2023. Following his surgery, the patient complained of difficulty opening his jaw, difficulty speaking clearly, and difficulty carrying out basic daily functions like drinking water and eating. A referral for physiotherapy was made for the patient's complaint. Timeline is shown in Table 1.

**Table 1:** Timeline

Dates	Events
21/3/23	The patient was admitted
22/3/23	Diagnosed and planned for surgery
31/3/23	Operation done
02/4/23	Referred date to physiotherapy
02/4/23	Started physiotherapy

Clinical findings

The patient was educated about the condition and treatment, and his consent was obtained for the evaluation and treatment. The user usability of the VR apparatus was checked and it was 75. The patient was assessed in a lower-lying position with the head end elevated to 30 degrees. His neck was slightly flexed, his shoulder was protracted, his elbow and wrist were in extension bilaterally, and his bilateral hip, knee, and ankle joints were in extension. No abnormality was noticed. He was afebrile during the general examination; his blood pressure was 128/80 mmHg, his pulse was 86 beats per minute, and his respiration rate was 18 beats per minute. And there was Ryle's tube.

Pain Examination

**Figure 1:**
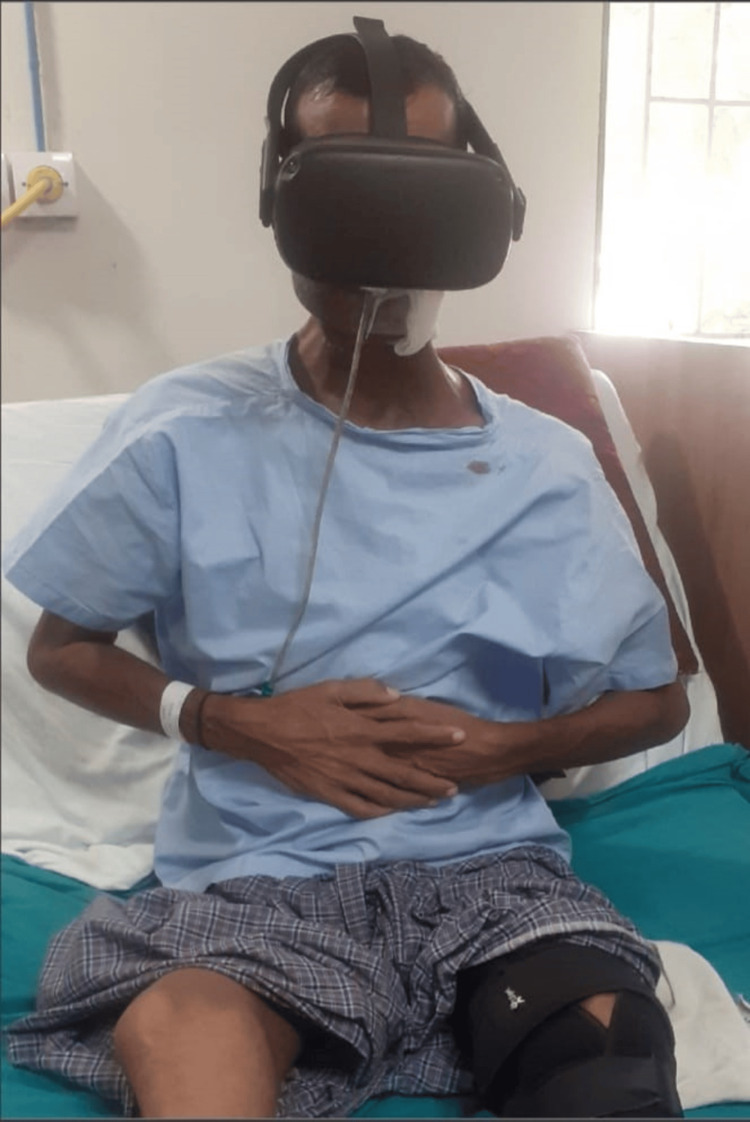
Virtual reality with deep breathing exercise

The patient winced in pain and had a grade of 3 tenderness. On the Numerical Pain Rating Scale, the scores for rest was 7 and movement was 9 (out of 10). Range of motion is shown in Table 2, manual muscle testing in Table 3, postoperative medication in Table 4, goal-oriented physiotherapy protocol in Table 5, and VR with deep breathing exercise in Figure 1.

**Table 2:** Range of motion

Variable	Movement	Range of motion
Cervical joint	Flexion	0-30°
	Extension	0-30°
	Lateral flexion (right)	0-20°
	Lateral flexion (left)	0-20°
Temporomandibular joint	Mouth opening	1.5 cm

**Table 3:** Manual muscle testing (according to MRC grading out of 5) MRC: Medical Research Council

Joint	Muscle	Left	Right
Cervical joint	Flexor	3	3
	Extensor	3	3
	Lateral flexor	3	3
Shoulder joint	Flexor	4	4
	Extensor	4	4
	Abductors	4	4
	Adductors	4	4
	Medial rotators	4	4
	Lateral rotators	4	4

**Table 4:** Postoperative medications NS: normal saline; IV: intravenous; SC: subcutaneous; PCM: paracetamol; IU: international unit; HS: hora somni (at night); OPD: outpatient department; mg: milligram; Inj: injection; Tab: tablet; g: gram

Medication
Inj. tramadol 50 mg in 100 NS IV stat
Inj. heparin 5000 IU SC twice daily×5 days
Inj. ceftriaxone 1 g IV twice daily×7 days
Inj. Pan 40 mg (Alkem Laboratories, Mumbai, India) IV once daily×7 days
Inj. PCM 1 g in 100 mg IV twice daily×2 days
Inj. Chymoral Forte (Torrent Pharmaceuticals, Ahmedabad, India) twice daily×7 days
Tab. Supradyn (Bayer, Berlin, Germany) once daily×10 days
Tab. Limcee 500 mg (Abbott Healthcare Pvt Ltd, Himachal Pradesh, India) twice daily×15 days
Tab. Ecosprin 75 mg (USV, Mumbai, India) HS from OPD day 4

**Table 5:** Goal-oriented physiotherapy protocol for two weeks Reps: repetitions; ROM: range of motion; SLR: straight leg raise

Sr. no.	Physiotherapy goals	Therapeutic intervention	Treatment regimen
1	To enlighten the patient and her family regarding her present medical condition	The importance of a carefully thought-out rehabilitation and fitness program should have been explained to the patient and their family	Early ambulation, positioning, and resumption of activities of daily life education
2	To initiate mouth opening	Active ROM for the temporomandibular joint: mouth opening and closing, jaw deviations	10 reps×3 times/day
		Chin tucks exercise	10 reps×3 times/day
3	To improve the ROM of the cervical joint	Flexion, extension, lateral flexion, and rotation of the cervical joint	Each movement 10 reps×3 times/day
4	To maintain the strength of the upper limb	Exercising the upper body while holding a 0.5-liter water bottle	10 reps×3 times/day
		Cervical isometric strengthening exercises	10 reps×3 times/day
5	To keep the lung's ability to function in good condition	Diaphragmatic breathing	10 reps×3 times/day
		Thoracic expansion exercise (3-second hold)	10 reps×3 times/day
6	To prevent secondary complications (deep vein thrombosis, pressure sores)	Ankle pumps	10 reps×3 times/day
		Dynamic quadriceps	10 reps×3 times/day
		SLR	10 reps×3 times/day
7	Functional mobility	Spot marching	10 reps×3 times/day
		Ambulation	For 10 minutes

VR for stress, anxiety, and depression

Mechanisms

The VR experience is expertly designed for Oculus Quest headsets through the integration of the Oculus SDK. The meticulously planned mechanics, which incorporate interactive breathing elements such as pursed lip and diaphragmatic breathing, augment the overall therapeutic effect.

Dynamics

To guarantee continuous patient engagement, the guided VR experience introduces increasingly difficult breathing exercises and leads patients through a structured progression from orientation to complex interactive elements.

Aesthetics

The film of the natural settings, which includes placid lakes and woodlands, creates a soothing atmosphere, and the visually appealing Oculus Quest headsets greatly enhance the overall good user experience.

Intervention

The two-week program, which consists of daily 20-minute VR sessions, creates a structured timeline for evaluating the long-term effects. A dynamic loop for continuous improvement is formed by regular evaluation using the System Usability Scale (SUS) and user feedback sessions.

Outcome measures

The Depression, Anxiety, and Stress Scale-21 (DASS-21) scoring* *before and after the use of VR is given in Table 6.

**Table 6:** DASS-21 scoring before and after the use of virtual reality DASS-21: Depression, Anxiety, and Stress Scale-21; POD: postoperative day

DASS	POD 5	POD 15
Depression	8	4
Anxiety	9	3
Stress	16	6

Home exercise program

On April 15, 2023, the patient was discharged from care. As part of a home exercise program, he was asked to keep up all his workouts. He received the following exercise advice as well: bubble blowing exercise, water holding exercise (the patient was instructed to swish water around in his mouth for 10 seconds before letting go), self-resisting exercise for the mouth (the patient was instructed to open his mouth and place his thumb on one side, then close it, and direct it to the other side, holding for 10 seconds before releasing).

## Discussion

Over the past 20 years, emerging technologies have found numerous uses in the field of healthcare, offering both immersive and non-immersive experiences. One such technology is VR, which immerses users in a three-dimensional (3D) world on a computer [15]. Multiple studies have revealed that a sedentary lifestyle is currently one of the serious health issues connected to several diseases, including cancer, metabolic syndrome, hypertension, heart problems, and psychological disorders [16]. Several psychological disorders, including various anxiety and depression disorders, have been successfully treated with VR therapy [17]. The main benefit of VR is that it provides an encouraging immersive environment and stimulates multiple senses [18]. It can effectively augment and enhance traditional treatment methods, such as exposure and cognitive behavioral therapy [19]. Immersive VR stimulates as many senses as possible to lessen the perception of pain, stress, anxiety, and other side effects during various medical procedures, including chemotherapy, in cancer patients. This is done by altering awareness and sensitivity to stressors, refocusing attention, concentration, and emotional input, rerouting neural signals, and substituting other neutral or enjoyable events for harmful stimuli [16].

Psychological instability might be one of the variables which lengthen a patient's hospital stay during treatment or increases the amount of sedation needed during a painful procedure. Additionally, a stressed patient struggles to cooperate with medical professionals and does not tolerate treatment well, which makes it challenging to complete the process [20].

VR is widely used in a wide range of medical applications, such as surgery, pain and anxiety management, treating phobias and addictions, fitness, gait training, weight loss, and therapeutic interventions for conditions like burns, breast cancer, pulmonary conditions like chronic obstructive pulmonary disease, and dental and abdominal surgeries, and it effectively improves patient experiences and outcomes across a spectrum of healthcare domains [21-23].

With the increasing number of cancer survivors and the well-documented rates of disability, cancer rehabilitation is an important component of cancer patient care, becoming more and more crucial [24]. Using landscape video in VR, it was found that patients with oral cancer experienced reduced levels of anxiety, stress, and depression.

## Conclusions

Patients report feeling incredibly at ease and relieved after watching our VR landscape video. Stress and anxiety can be reduced in a therapeutic environment created by the calming imagery, calming music, and relaxing meditation. Patients experience a greater sense of connectedness to both the supportive community of other patients and the virtual environment. Patients with oral cancer frequently and seriously experience anxiety and depressive symptoms simultaneously, which worsens prognosis and raises mortality in this population of patients. The addition of immersive VR therapy to oral rehabilitation helps patients with oral cancer by enhancing their mood and lowering their anxiety and stress levels. To determine whether the effects obtained during the stay in the rehabilitation ward are sustainable, additional follow-up studies with long-term observation of patients are required. The above study suggests that early physiotherapy rehabilitation helps improve a patient's quality of life. A key component of a successful recovery is the reduction of stress and anxiety achieved through VR therapy with landscape video.
